# Role of TRPC1 in the pathogenesis of depression induced by traumatic brain injury

**DOI:** 10.3389/fnins.2026.1774265

**Published:** 2026-04-16

**Authors:** Qi-Hang Pan, Lin-Han Li, Ming-Bo Fan, Yu Xia, Xiao-Long Liu, Zhen-Huan Chen, Fei Sun, Ting He, Qiu-Zhi Zhou, Meng-Zhu Li, Jun Li

**Affiliations:** 1 Department of Neurosurgery, The Central Hospital of Wuhan, Tongji Medical College, Huazhong University of Science and Technology, Wuhan, China; 2 Key Laboratory for Molecular Diagnosis of Hubei Province, The Central Hospital of Wuhan, Tongji Medical College, Huazhong University of Science and Technology, Wuhan, China; 3 Department of Neurosurgery, Tongji Hospital of Tongji Medical College of Huazhong University of Science and Technology, Wuhan, Hubei, China; 4 Maternal and Child Health Hospital of Hubei Province, Wuhan, Hubei, China; 5 Department of Pathophysiology, School of Basic Medicine, Key Laboratory of Education Ministry of China/Hubei Province for Neurological Disorders, Tongji Medical College, Huazhong University of Science and Technology, Wuhan, China; 6 Department of Rehabilitation, Tongji Hospital, Tongji Medical College, Huazhong University of Science and Technology, Wuhan, Hubei, China

**Keywords:** depression, inflammatory cytokines, synaptic impairment, traumatic brain injury, TRPC1

## Abstract

**Background:**

Traumatic brain injury (TBI) is one of the leading causes of mortality and disability, with many patients developing long-term sequelae. Depression is among the most common psychiatric complications following TBI, yet its underlying mechanisms remain unclear. Transient receptor potential canonical 1 (TRPC1) has been implicated in neurological disorders, but its role in post-TBI depression is not well understood.

**Methods:**

A controlled cortical impact (CCI) model was used to induce moderate TBI in mice. At 4 weeks post-injury, depressive-like behaviors were assessed using the tail suspension test (TST), forced swim test (FST), and sucrose preference test (SPT). Subsequently, reactive astrocytes and microglia were quantified, along with the expression of inflammatory cytokines, in the ipsilateral hippocampus. Synaptic function was also evaluated.

**Results:**

Behavioral tests revealed that TBI mice exhibited significant depressive- and anxiety-like behaviors at 4 weeks post-injury. Concurrently, TRPC1 expression was downregulated in the ipsilateral hippocampus, accompanied by reduced levels of synaptic-associated proteins, elevated pro-inflammatory cytokines, and increased reactive astrocytes and microglia. Further experiments demonstrated that TRPC1 overexpression attenuated neuroinflammation, restored synaptic function, and ameliorated depressive-like behaviors in TBI mice.

**Conclusion:**

This study suggests that TBI may trigger depression by downregulating TRPC1, thereby promoting neuroinflammation and synaptic dysfunction. Conversely, TRPC1 overexpression mitigates these effects, highlighting its potential as a therapeutic target for post-TBI depression.

## 1 Introduction

Traumatic brain injury (TBI) represents the most prevalent neurological disorder worldwide and stands as a leading cause of mortality and long-term disability, imposing a substantial burden on public health system (Bell et al., 2017; Jiang et al., 2019; Maas et al., 2017; Ye et al., 2024). Importantly, TBI is not merely an acute condition but rather a chronic disease with progressive consequences, significantly elevating the risks of psychiatric disorders and delayed-onset neurodegenerative diseases (Gao et al., 2020; Maas et al., 2022; McAllister, 2008; Zahniser et al., 2019). Clinical evidence demonstrates that TBI patients face markedly increased susceptibility to neuropsychiatric disturbances compared to healthy populations, frequently developing severe psychiatric manifestations including depression, post-traumatic stress disorder (PTSD), and manic episodes (Drange et al., 2018; Mikolić et al., 2025; Monsour et al., 2022). This epidemiological pattern underscores the critical need to investigate the pathophysiological mechanisms linking TBI to subsequent neuropsychiatric morbidity, particularly given the limited efficacy of conventional antidepressants in this patient population (Albrecht et al., 2020; Passler et al., 2022; Stein et al., 2019).

The pathological manifestations of TBI comprise two distinct yet interrelated components: primary and secondary injuries (Orr et al., 2024; Volpe, 2009). Primary injury results from immediate mechanical damage induced by external forces, leading to skull fractures, intracranial hematomas, and direct neuronal death (Banoei et al., 2025; Pearn et al., 2017). Secondary injury evolves hours to months post-trauma through complex pathophysiological cascades, including dysregulated immune responses, calcium overload, excitotoxic neurotransmitter release, and oxidative stress, collectively exacerbating cerebral ischemia, edema, and intracranial hypertension (Fesharaki-Zadeh, 2022; Hegdekar et al., 2023; Sulhan et al., 2020). Notably, persistent secondary injury mechanisms critically impair neuroprotective and reparative processes, resulting in delayed and incomplete neurological recovery (Jassam et al., 2017; Simon et al., 2017b). Among these mechanisms, neuroinflammation mediated by microglial activation has emerged as a central driver of both acute neuronal damage and chronic functional impairments post-TBI (Corps et al., 2015). Preclinical evidence demonstrates that microglia perpetuate long-term neuropathological changes, with microglial depletion studies showing significant prevention of TBI-induced cognitive deficits (Witcher et al., 2021). Clinical and translational research further reveals elevated peripheral and central nervous system levels of pro-inflammatory cytokines (TNF-α, IL-1β, and IL-6) in both patients and animal models exhibiting depressive phenotypes after suffering TBI (Risbrough et al., 2022; Witcher et al., 2021). Collectively, these results indicate that neuroinflammatory signaling pathways are critically involved in the pathogenesis of post-traumatic depression-like behaviors.

TRPC1 primarily functions as a key regulator of store-operated calcium entry (SOCE), a critical process governing embryonic neural stem cell self-renewal, proliferation, and apoptosis (Wang et al., 2018; Zeng et al., 2021). The mechanistic cascade involves endoplasmic reticulum calcium depletion triggering STIM1 (stromal interaction molecule 1) oligomerization, which subsequently recruits and gates Orai1/TRPC1 channel complexes at ER-plasma membrane junctions to modulate intracellular Ca^2+^dynamics and downstream signaling (Ambudkar et al., 2017; Wang et al., 2020). Notably, in LPS-activated macrophages, caspase-11-mediated TRPC1 degradation enhances IL-1β secretion, while TRPC1 knockdown in neutrophils impairs chemotaxis and aggregation, which processes essential for inflammatory resolution (Py et al., 2014).

The aforementioned studies suggest that inflammatory responses may play a critical role in the pathogenesis of depression following TBI, but the precise regulatory mechanisms remain unclear. This study aims to investigate whether TRPC1 can modulate neuroinflammation and thereby contribute to the development of post-TBI depression, while exploring its potential as a novel therapeutic target for TBI-induced depressive disorders.

## 2 Materials and methods

### 2.1 Animals and grouping

The experimental protocol of this study was approved by the Animal Care and Use Committee of Huazhong University of Science and Technology. All procedures were performed in strict accordance with the NIH Guidelines for the Care and Use of Laboratory Animals. Mice were housed in groups of 3–5 per cage, with pregnant mice individually housed. All animals were maintained in a temperature-controlled (24 ± 1 °C) and humidity-regulated (55 ± 2%) facility under a 12/12 h light–dark cycle, with a constant pressure differential of 5 Pa relative to ambient environment. Food and water were provided ad libitum. C57BL/6 mice were used in Experiments 1 and 3, and TRPC1 knockout (TRPC1^ko^) mice were used in Experiment 2. TRPC1 knockout mice were a kind gift from Dr. Lutz Birnbaumer (Laboratory of neurobiology, National Institute of Environmental Health of Science, Research Triangle Park, NC 27709). Three-month-old male TRPC1 knockout mice of 129/SvEv genetic background and age-matched wild-type mice kept under standard laboratory conditions.

Experimental Groups

Experiment 1: SHAM (*n* = 14), TBI (*n* = 17)

Experiment 2: TRPC1^ko^ (*n* = 6), wild type (*n* = 6)

Experiment 3: VEC + SHAM (n = 12), VEC + TBI (n = 12), TRPC1 + SHAM (*n* = 12), TRPC1 + TBI (*n* = 12).

### 2.2 Stereotactic viral injection

Mice were anesthetized by intraperitoneal injection of 1% pentobarbital sodium, 50 mg/kg for injection, and body temperature was maintained using an electric heating pad. According to the mouse brain atlas (2nd edition) by Paxinos George and Franklin Keith, the hippocampal CA1 region was targeted (coordinates: AP: −2.00 mm, ML: ± 1.1 mm, DV: −1.3 mm) using a stereotaxic apparatus. A micro-drill was used to create a craniotomy above the target area, and the virus was delivered via a microsyringe (injection rate: 100 nL/min, total volume: 1000 nL, AAV-syn-TRPC1-eGFP, or the control AAV-syn-eGFP). After completion of the injection, the needle was left in place for 8 min before slow withdrawal. The scalp was sutured, and unconscious mice were placed on a 37 °C heating pad until full recovery from anesthesia before being returned to their home cages.

### 2.3 Moderate TBI model

Anesthesia was induced by intraperitoneal injection of 1% pentobarbital sodium, 50 mg/kg for injection, and body temperature was maintained using an electric heating pad. Under stereotaxic guidance, the hippocampal CA1 region was targeted (coordinates: AP: −2.00 mm, ML: ±1.1 mm, DV: −1.3 mm). A micro-drill was used to create a 3 cm^2^ cranial window while preserving the dura mater intact. In the traumatic brain injury (TBI) group, a cortical impact was delivered using a controlled cortical impact device (Wuhan Yihong Technology Co., Ltd.) with a 3-mm diameter impactor (velocity: 3.5 m/s, dwell time: 40 ms, depth: 1.3 mm) (Long et al., 2020). The scalp was then sutured. Sham-operated (SHAM) mice underwent identical craniotomy procedures without cortical impact. Unconscious mice were placed on a 37 °C heating pad until full recovery from anesthesia, after which neurological function was assessed using a standardized scoring system called Neurological Severity Score (NSS) (Supplementary Table S1) (Flierl et al., 2009).

### 2.4 Behavior testing

Behavioral tests were performed at the specified time points (4 weeks post-TBI) according to the experimental protocol. Mice underwent the open field test (OPT), tail suspension test (TST), forced swimming test (FST), and sucrose preference test (SPT) sequentially. To minimize interference between tests, mice were housed individually for 1 day to recover before the next assessment.

#### 2.4.1 Open field test (OPT)

Prior to testing, the arena was virtually divided into 16 equal squares (10 cm × 10 cm) using behavioral analysis software. The central zone was defined as the combined area of four central squares, while the peripheral zone comprised the four corner squares. During the 5-min test, parameters recorded included, (1) Total distance traveled in the arena, (2) Number of entries into the central zone, (3) Time spent in the central zone.

#### 2.4.2 Tail suspension test (TST)

Mice were suspended by the distal third of their tails using adhesive tape on a horizontal bar (15 cm above the ground). During the 6-min test, the latency to the first immobility episode and the total immobility time during the last 4 min were recorded. Immobility was defined as passive hanging without movement; struggling was characterized by active body curling (Vicentini et al., 2021).

#### 2.4.3 Forced swimming test (FST)

Mice were placed in a transparent cylindrical tank (20 cm diameter × 40 cm height) filled with water (25 cm depth, 25 ± 1 °C). During the 6-min test, the latency to the first immobility episode and the total immobility time during the last 4 min were recorded. Immobility was defined as floating motionless; struggling was identified by vigorous paddling using the head and forepaws (Saito et al., 2021).

#### 2.4.4 Sucrose preference test (SPT)

Mice were acclimated to 1% sucrose solution (100 mL) for 24 h. Subsequently, they had free access to both 1% sucrose and plain water for 24 h, with bottle positions switched every 12 h during habituation to eliminate side bias. After 24 h of food deprivation, sucrose and water consumption were measured over 8 h. Bottles were identical and randomly positioned. Sucrose preference (%) was calculated as:

Sucrose Preference = Sucrose intake/(Sucrose intake + Water intake) × 100% (Liu et al., 2018).

### 2.5 Animal euthanize and sample collection

After the behavioral tests, the mice were anesthetized by intraperitoneal injection of 1% pentobarbital sodium (50 mg/kg) prior to specimen collection according to the experimental requirements. For Western blot analysis, mice were perfused with ice-cold phosphate-buffered saline (PBS), and hippocampal tissues were rapidly dissected on ice before storage at −80 °C. For histochemical and immunofluorescence staining, mice were transcardially perfused with 4% paraformaldehyde (PFA) in PBS. The whole brain was post-fixed, dehydrated in 30% sucrose, embedded in OCT compound, and sectioned coronally at 40 μm thickness using a pre-cooled cryostat maintained at −20 °C. Sections were stored in cryoprotectant solution at 4 °C until further processing.

### 2.6 Immunofluorescence

Immunofluorescence staining was performed to evaluate GFAP and Iba-1 intensity. Brain sections at matched or adjacent levels were selected, permeabilized with 0.5% Triton-X100 in 1 × PBS, and blocked with 5% BSA for 30 min. Primary antibodies were applied overnight at 4 °C, followed by incubation with secondary antibodies for 1 h at room temperature. Nuclei were counterstained with Hoechst for 30 min. Sections were mounted on non-adhesive slides and coverslipped with 50% glycerol in 1 × PBS. Images were acquired using a two-photon confocal laser-scanning microscope, and quantitative analysis was performed with ImageJ software (NIH, United States). Regions of interest were defined, and images were converted to 8-bit grayscale. A consistent threshold was applied to distinguish positive staining. The “Analyze Particles” function was used to count cells with size exclusion set to 10–100 μm^2^ to exclude debris.

### 2.7 Immunohistochemistry

Immunohistochemical staining was performed to assess GFAP and Iba-1 expression intensity. Coronal brain sections at matched or comparable levels were selected and treated with 3% H_2_O_2_ in PBS to quench endogenous peroxidase activity. Sections were then permeabilized with 0.5% Triton-X100 in 1 × PBS and blocked with 5% BSA for 45 min at room temperature. Primary antibodies were applied and incubated overnight at 4 °C, followed by incubation with appropriate secondary antibodies for 1 h at room temperature. The stained sections were mounted on adhesive slides, dehydrated through a graded ethanol series, cleared in xylene, and cover slipped with neutral resin mounting medium.

### 2.8 Golgi staining and spine analysis

Golgi staining was performed by using a FD Rapid Golgi Stain Kit (FD Rapid GolgiStain TM Kit, PK401). After anesthetized, the brain of mouse was soaked in the AB mixture solution for 24 h. The brain was placed into new AB solution and soaked for 1 month. During this period, it was gently shaken twice a week. One month later, the brain was placed into C solution and soaked for 3–7 days, sliced, 100 μm, patched, and dried. The brain slice was stained by follows: washed with ddH2O 2 × 4 min; soaked by mixture (D solution: E solution: ddH2O = 1:1:2) for10min; washed with ddH2O for 2 × 4 min; dehydrated in 50, 75, and 95% alcohol gradients for 4 min each, then absolute ethanol 4 × 4 min; xylene 3 x 4 min, left for a few hours, sealed. The slice were imaged with an optical microscope, counted as described previously (Wang et al., 2021).

### 2.9 Nissl staining

Nissl staining was performed to identify neuronal cytoarchitecture in brain sections. Following mounting onto adhesive slides, the brain sections were subjected to Nissl staining for 10 min. Decolorization was then performed using 75% ethanol for approximately 5 min, followed by sequential dehydration in graded ethanol solutions of 80, 90, and 100% for 2 min each. The durations of staining and decolorization were adjusted dynamically based on the staining intensity observed under microscopic examination. After dehydration, the sections were cleared in xylene for 1 h and subsequently mounted with neutral balsam.

### 2.10 Western blot

Hippocampal tissues and cultured cells were collected for protein extraction. Protein concentrations were determined using a BCA assay kit. Protein samples were mixed with loading buffer (P0015F, Beyotime, China), heated at 95 °C for 5 min, and separated by SDS-PAGE. The separated proteins were then transferred onto nitrocellulose membranes. After blocking with 5% skim milk for 30 min, membranes were incubated with primary antibodies at 4 °C overnight, followed by incubation with secondary antibodies for 1 h at room temperature. Protein bands were visualized using an ECL chemiluminescence kit and imaged. Quantitative analysis of band intensity was performed using ImageJ software. Relative band densities were quantified using ImageJ software. Antibodies used in the present study include anti-PSD95 (Abcam, ab18258), anti-NR2A (Abcam, ab124913), anti-NR2B (Abcam, ab65783), anti-STIM1 (Sigma, S6072), anti-TRPC1 (Sigma, T8276), anti-TNF-α (Abcam, ab183218), anti-IL-1β (Abcam, ab234437), anti-SYN (Abcam, ab138501), and anti-β-ACTIN (Abcam, ab6272).

### 2.11 Quantitative real-time PCR (qRT-PCR)

The cells were washed with ice-cold PBS, and total RNA was extracted using TRIzol reagent (Cat# 15596026, Thermo Fisher, United States). Reverse transcription was performed using a cDNA synthesis kit (Vazyme) following the manufacturer’s protocol. Quantitative real-time PCR (qPCR) was conducted using a Vazyme SYBR Green Master Mix on a Roche LightCycler system (Roche, Switzerland). The relative expression of target genes was normalized to β-actin using the 2^−ΔΔCt^ method. The primer of *Tnfα*-forward was CCCTCACACTCAATCATCTTCT and *Tnfα*-reverse was GGCTACGACGTGGGCTACAG. The primer of *Il6*-forward was TGGGGCTCTTCAAAAGCTCC and *Il6*-reverse was AGGAACTATCACCGGATCTTCAA.

### 2.12 Statistical analysis

All data were collected and analyzed in a blinded manner. All investigators performing behavioral tests, histological analyses, and data quantification were blinded to experimental group assignments. Data were analyzed using Graphpad software. Student’s *t*-test was used for the comparison between two groups, the one-way ANOVA followed by Tukey’s multiple comparisons test was used to analysis the data among four groups. The results were presented as mean ± SEM and *p* < 0.05 was accepted as statistically significant.

## 3 Results

### 3.1 TBI induced depressive like behavior

**Figure 1:**
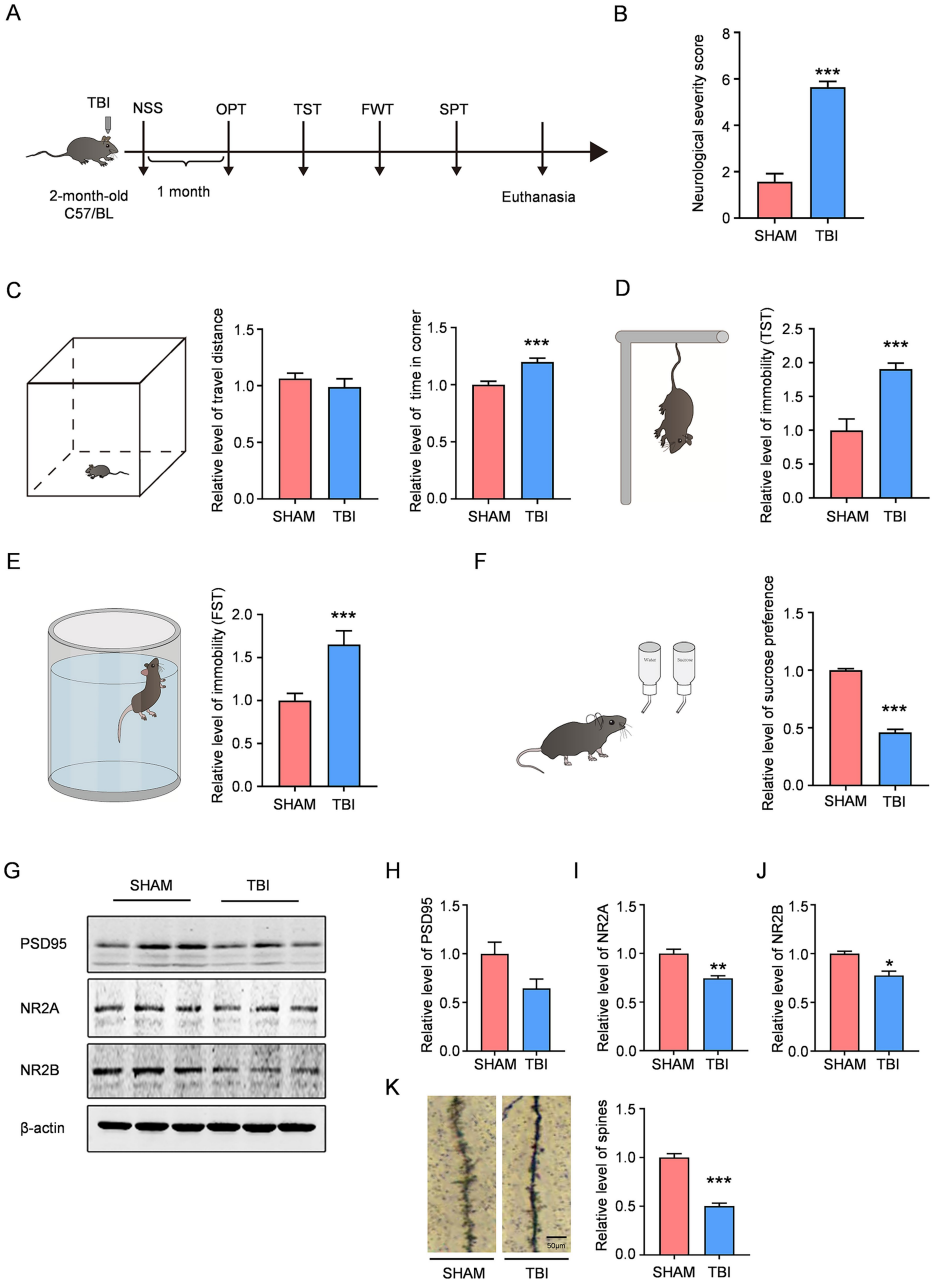
TBI induces depression-like behaviors and synaptic injury compared to the sham group: **(A)** 2 months male C57BL/6 mice were subjected to control (SHAM) or CCI (TBI). **(B)** Neurologic severity score (NSS) changes following traumatic brain injury, *n* = 14 for SHAM, *n* = 17 for TBI. **(C)** Travel distance and the time in corner of open field test of each group after 4 weeks following moderate TBI, *n* = 14 for SHAM, *n* = 17 for TBI. **(D)** The immobility time of TST after 4 weeks following moderate TBI, *n* = 14 for SHAM, *n* = 17 for TBI. **(E)** The immobility time of FST after 4 weeks following moderate TBI, *n* = 14 for SHAM, *n* = 17 for TBI. **(F)** The percentage of sucrose preference of each group after 4 weeks following moderate TBI, *n* = 14 for SHAM, *n* = 17 for TBI. **(G–J)** Western blotting showing the expression of PSD95 (*n* = 3 per group), NR2A (*n* = 3 per group), and NR2B (*n* = 3 per group) in hippocampus from the mice with and without TBI. **(K)** Golgi staining analysis of dendritic spine density in hippocampal neurons across experimental groups, *n* = 24 per group. Data are analyzed by Student’s *t*-test, represented as mean ± SEM, ***p < 0.001, **p < 0.01, *p < 0.05.

Two-month-old mice were subjected to cortical impact injury (CCI) to induce TBI. After recovery from anesthesia, the Neurological Severity Score (NSS) was assessed. The results showed that the TBI group exhibited moderate brain injury, with NSS significantly higher than that of the SHAM group, indicating significant neurological impairment in TBI mice (Figure 1B). To determine whether the mice developed depression-like phenotypes post-injury, behavioral tests were conducted 4 weeks after TBI (Figure 1A). The open field test revealed no significant difference in total distance traveled between the two groups, suggesting that motor function in TBI mice had recovered to normal levels by 4 weeks post-injury. However, TBI mice spent significantly more time in the corners compared to SHAM mice, indicating anxiety-like behavior (Figure 1C). The tail suspension test showed that immobility time was significantly longer in the TBI group than in the SHAM group (Figure 1D). Similarly, the forced swim test demonstrated a significant increase in immobility time in TBI mice (Figure 1E). The sucrose preference index, a key indicator of depressive behavior, was significantly reduced in TBI mice compared to SHAM controls (Figure 1F). These depression-related behavioral results collectively demonstrate that TBI mice exhibit significant depression-like behaviors at 4 weeks post-injury. Synaptic impairment is a hallmark of depression, with synaptic-related proteins often downregulated in depressive disorders. Western blot analysis revealed that the expression levels of synaptic proteins NR2A and NR2B were significantly decreased in the hippocampus of TBI mice compared to SHAM controls, while PSD95 expression showed a decreasing trend, suggesting synaptic damage in TBI-induced depressive mice (Figures 1G–J). Golgi staining showed that dendritic spine density was reduced in the TBI group compared to the SHAM group (Figure 1K). These findings indicate that TBI can induce depression-related manifestations in mice, though the underlying mechanisms require further investigation.

### 3.2 Traumatic brain injury leads to elevated hippocampal inflammation and decreased TRPC1 expression in mice

**Figure 2:**
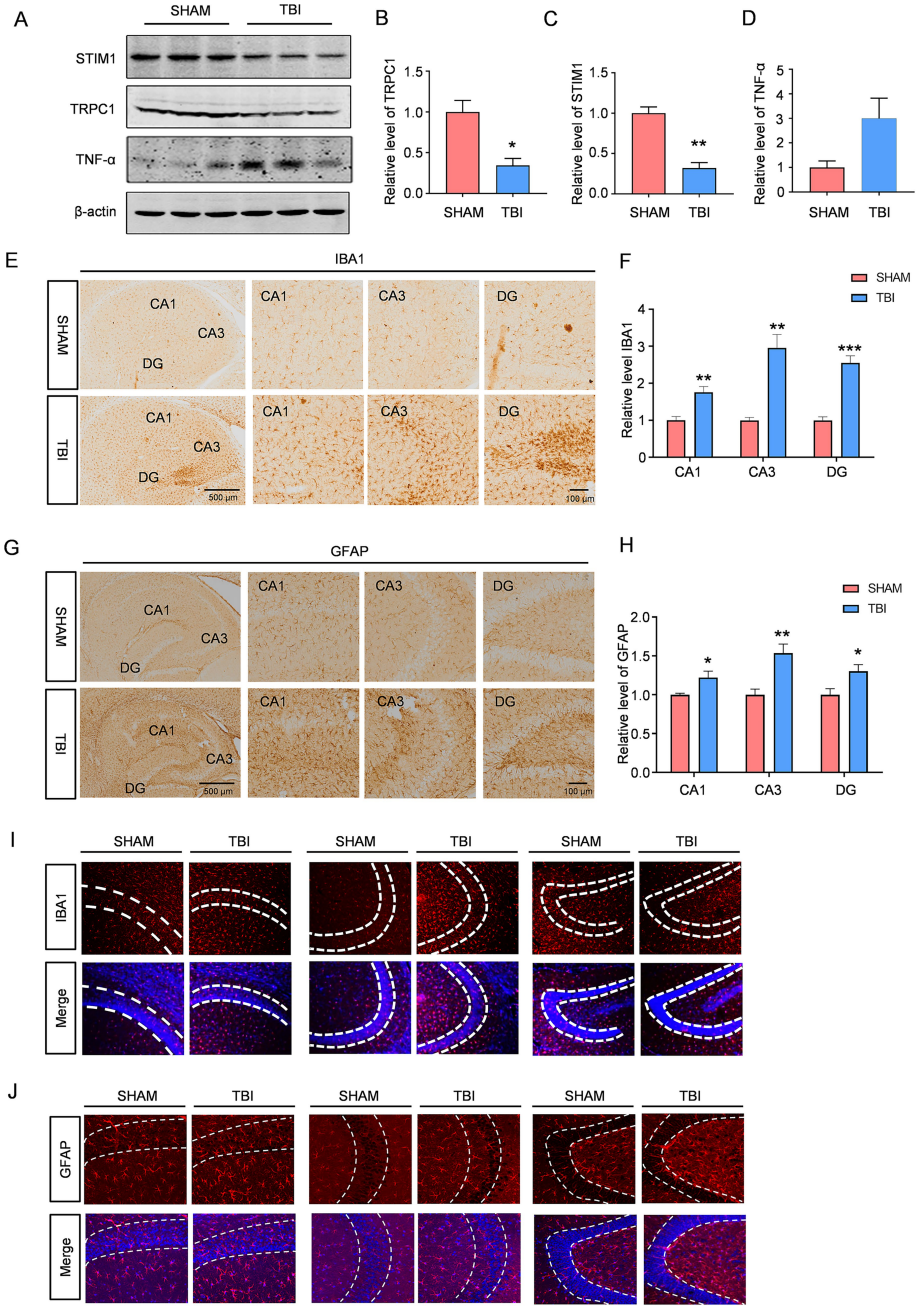
TRPC1/STIM1 expression is downregulated in the hippocampal region following TBI, accompanied by elevated neuroinflammation: **(A–D)** Western blotting showing the expression of TRPC1, STIM1, and TNF-*α* in hippocampus from the C57BL/6 mice with and without TBI, *n* = 3 per group. **(E,F)** The level of IBA1 in hippocampus from the C57BL/6 mice with and without TBI, were measured by immunofluorescence staining, *n* = 4 per group. **(G,H)** The level of GFAP in hippocampus from the C57BL/6 mice with and without TBI, were measured by immunofluorescence staining, *n* = 4 per group. **(I,J)** Immunohistochemistry staining reveals the level of IBA1 and GFAP in hippocampus from the C57BL/6 mice with and without TBI. Data are analyzed by Student’s *t*-test, represented as mean ± SEM; ***p < 0.001, **p < 0.01, *p < 0.05.

Studies suggest that chronic neuroinflammation occurs following TBI and may contribute to the development of depression (Bodnar et al., 2018; Postolache et al., 2020). Western blot analysis revealed that the expression of the pro-inflammatory cytokine TNF-α was significantly elevated in the hippocampus of TBI mice compared to SHAM controls, indicating enhanced neuroinflammation in TBI-induced depressive mice (Figures 2A–D). Histochemical and immunofluorescence staining further demonstrated a marked increase in GFAP (a marker of astrocytes) and IBA1 (a marker of microglia) expression in the hippocampal CA1, CA3, and dentate gyrus (DG) regions of TBI mice, suggesting reactive astrogliosis and microglial activation (Figures 2E–J). Given that TRPC1 has been implicated in the regulation of inflammatory responses (Chen et al., 2021; Wu et al., 2023), we investigated whether TRPC1 plays a role in post-TBI depression. Western blot analysis showed that the protein levels of TRPC1 and its interacting partner STIM1 were significantly reduced in the hippocampus of TBI mice (Figures 2A,B). These findings suggest that downregulation of TRPC1/STIM1 signaling may contribute to the pathogenesis of post-TBI depression, possibly through the modulation of neuroinflammatory processes.

### 3.3 TRPC1 modulates inflammatory responses and its deficiency induces depression-like behaviors

**Figure 3:**
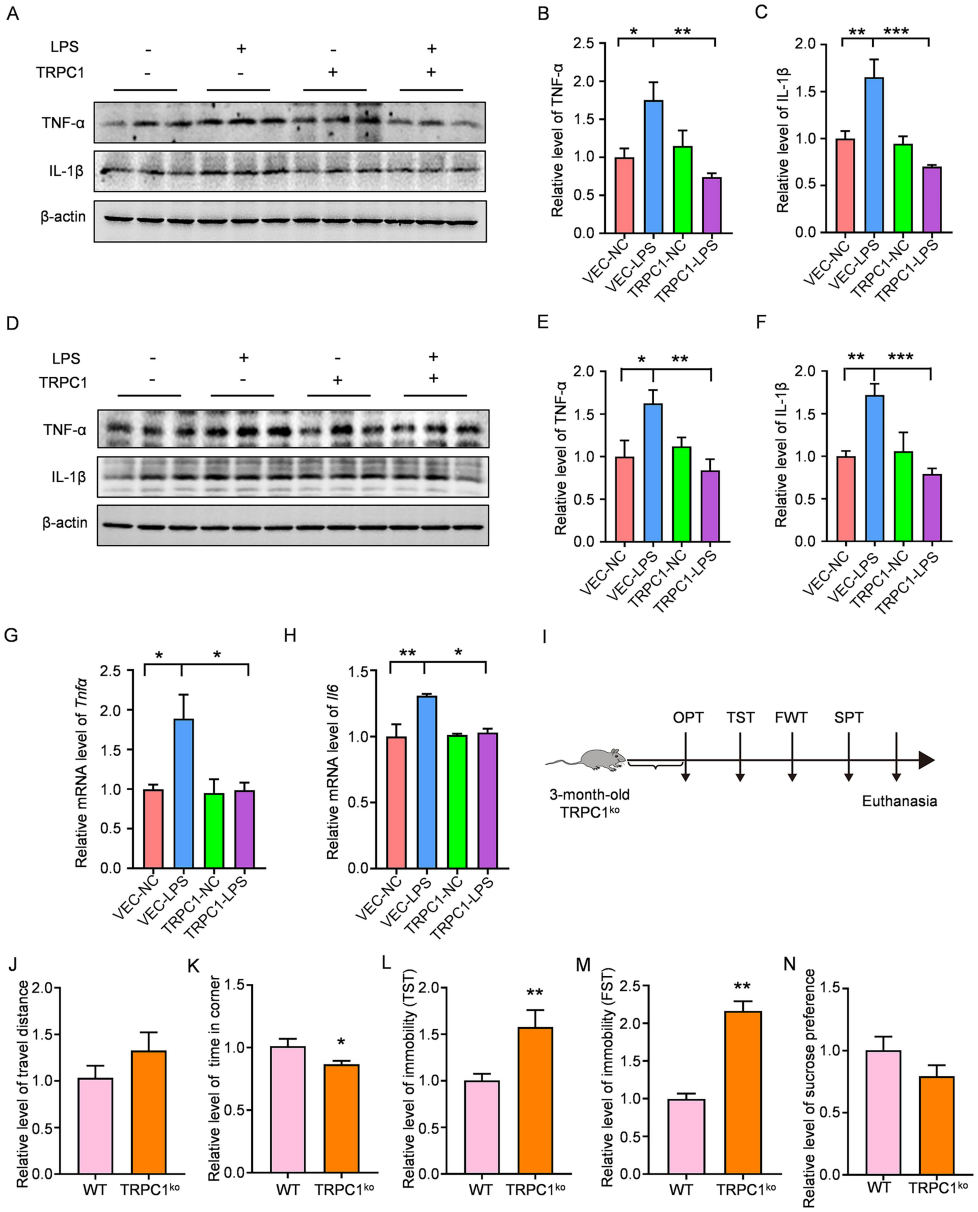
TRPC1 overexpression attenuates LPS-induced inflammatory responses: **(A–C)** HEK293 cells were transfected with TRPC1 overexpression plasmid and stimulated with LPS. Protein expression levels of TNF-α (*n* = 6 per group) and IL-1β (*n* = 6 per group) were measured by Western blot, *n* = 3 per group, *p* < 0.001, *F* = 7.732. **(D–F)** N2a cells overexpressing TRPC1 were treated with LPS, followed by WB detection of TNF-α (*n* = 6 per group) and IL-1β (*n* = 6 per group) protein levels, *n* = 3 per group, *p* < 0.001, *F* = 5.171. **(G,H)** qPCR analysis of *Tnfα* (*n* = 6 per group) and *Il6* (*n* = 3 per group) mRNA expression in TRPC1-overexpressing N2a cells after LPS treatment. **(I)** Schematic timeline of behavioral tests performed in 3-month-old TRPC1^ko^ and WT mice, *n* = 3 per group, *p* < 0.01, *F* = 7.449. **(J,K)** Comparison of total locomotion distance and time spent in corner zones between TRPC1^ko^ and WT mice in the OFT, *n* = 6 per group. **(L)** Immobility duration of TRPC1^ko^ and WT mice in the TST, *n* = 6 per group **(M)** Immobility duration of TRPC1^ko^ and WT mice in the FST, *n* = 6 per group. **(N)** Sucrose preference ratio (%) of TRPC1^ko^ versus WT mice in the SPT, *n* = 6 per group. Data are analyzed by ne-way ANOVA with Tukey’s multiple comparisons test for **(A–H)**, by Student’s *t*-test for **(J–N)**, and represented as Mean ± SEM; ***p < 0.001, **p < 0.01, *p < 0.05.

To further investigate the regulatory role of TRPC1 in inflammation, we overexpressed TRPC1 in HEK293 cells and stimulated them with lipopolysaccharide (LPS) to induce inflammatory responses. Western blot analysis revealed that, compared to the vector control (VEC-NC) group, LPS treatment (VEC-LPS) significantly increased the protein levels of TNF-α and IL-1β. However, TRPC1 overexpression (TRPC1-LPS) markedly attenuated this LPS-induced upregulation of pro-inflammatory cytokines (Figures 3A–C), suggesting that TRPC1 exerts anti-inflammatory effects. To determine whether TRPC1 similarly regulates neuroinflammation, we overexpressed TRPC1 in N2a neuronal cells and treated them with LPS. Consistent with the findings in HEK293 cells, LPS stimulation (VEC-LPS) significantly elevated TNF-α and IL-1β protein levels compared to the control (VEC-NC), whereas TRPC1 overexpression (TRPC1-LPS) suppressed this increase (Figures 3D–F). Furthermore, qPCR analysis demonstrated that LPS-induced upregulation of *Tnfα* and *Il6* mRNA levels was significantly reduced in TRPC1-overexpressing cells (Figures 3G,H), further supporting the anti-inflammatory role of TRPC1 in neural cells.

To assess whether TRPC1 deficiency contributes to depression-like behaviors, we subjected 3-month-old TRPC1^ko^ mice to a series of behavioral tests (Figure 3I). The open field test revealed no significant difference in total distance traveled between TRPC1^ko^ and WT mice, indicating normal locomotor function. However, TRPC1^ko^ mice spent significantly more time in the corners, suggesting increased anxiety-like behavior (Figures 3J,K). In the TST and forced FST, TRPC1^ko^ mice exhibited significantly longer immobility times than WT controls (Figures 3L,M), indicating heightened despair-like behavior. Additionally, the sucrose preference test showed a decreasing trend in the preference index in TRPC1^ko^ mice (Figure 3N), further supporting a depression-like phenotype.

### 3.4 Overexpression of TRPC1 ameliorates depression-like behaviors in mice after traumatic brain injury

**Figure 4:**
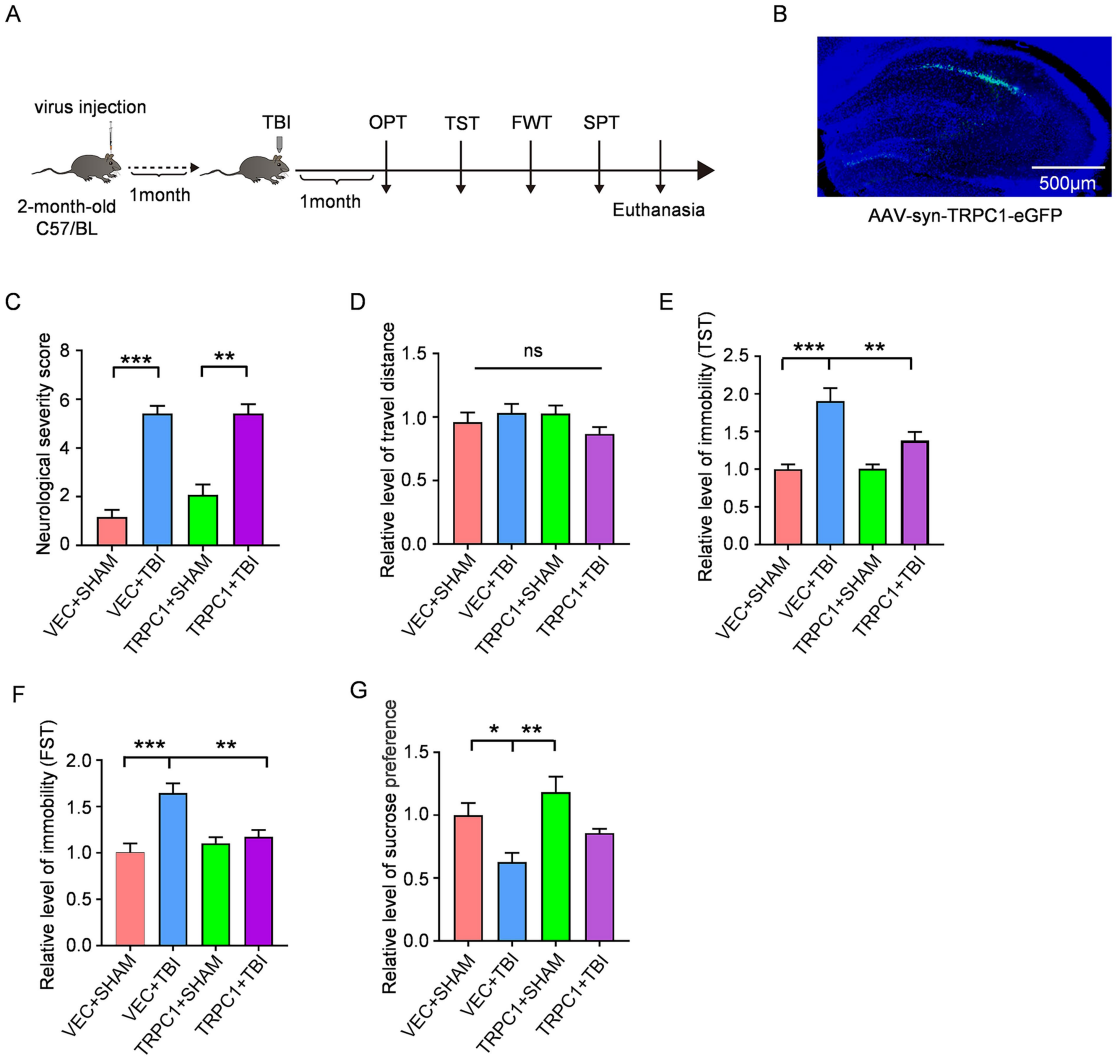
TRPC1 overexpression in hippocampus attenuates the depressive-like behaviors induced by TBI: **(A)** Schematics of the experiment procedure: Two-month-old mice received intracranial injection of TRPC1-overexpressing viral vectors. After 4 weeks of viral expression, TBI was induced, and followed by NSS. Behavioral tests for depression (OFT, TST, FST, and SPT) were performed 4 weeks post-TBI. **(B)** The fluorescence images of mice overexpressing TRPC1. **(C)** Neurological severity scores following traumatic brain injury, *n* = 12 per group. **(D)** No significant difference in locomotor activity was observed at 4 weeks post-TBI, *n* = 12 per group. **(E)** Immobility time in the TST, *n* = 12 per group. **(F)** Immobility time in the FST, *n* = 12 per group. **(G)** Sucrose preference in the SPT, *n* = 6 per group. Data are analyzed by One-way ANOVA with Tukey’s multiple comparisons test, represented as mean ± SEM; ***p < 0.001, **p < 0.01, *p < 0.05.

To further investigate whether modulation of TRPC1 could serve as a potential therapeutic target for post-TBI depression, lentivirus-mediated TRPC1 overexpression was performed in 2-month-old mice. One month later, CCI was conducted to induce TBI (Figures 4A,B). The NSS assessed after anesthesia recovery showed that the TBI group exhibited moderate brain injury, with significantly higher NSS compared to the SHAM group (Figure 4C), indicating significant neurological impairment in TBI mice. To determine whether TRPC1 overexpression alleviates depression-like behaviors, behavioral tests were performed 4 weeks post-TBI. The open field test revealed no significant differences in total travel distance among the four groups, suggesting that locomotor function in TBI mice had recovered to baseline levels by 4 weeks post-injury (Figure 4D). In the TST, the VEC-TBI group showed significantly increased immobility time compared to the VEC-SHAM group, whereas the TRPC1-TBI group exhibited markedly reduced immobility time relative to the VEC-TBI group (Figure 4E). Similarly, the FST demonstrated that the VEC-TBI group had prolonged immobility time compared to the VEC-SHAM group, while the TRPC1-TBI group showed significantly shortened immobility time versus the VEC-TBI group (Figure 4F). The SPT revealed a significant decrease in sucrose preference in the VEC-TBI group compared to the VEC-SHAM group, whereas the TRPC1-TBI group displayed an increasing trend in sucrose preference relative to the VEC-TBI group (Figure 4G). These behavioral results collectively demonstrate that TRPC1 overexpression ameliorates depression-like behaviors in mice following traumatic brain injury.

### 3.5 Overexpression of TRPC1 reduces neuroinflammation and improves synaptic function after traumatic brain injury in mice

**Figure 5:**
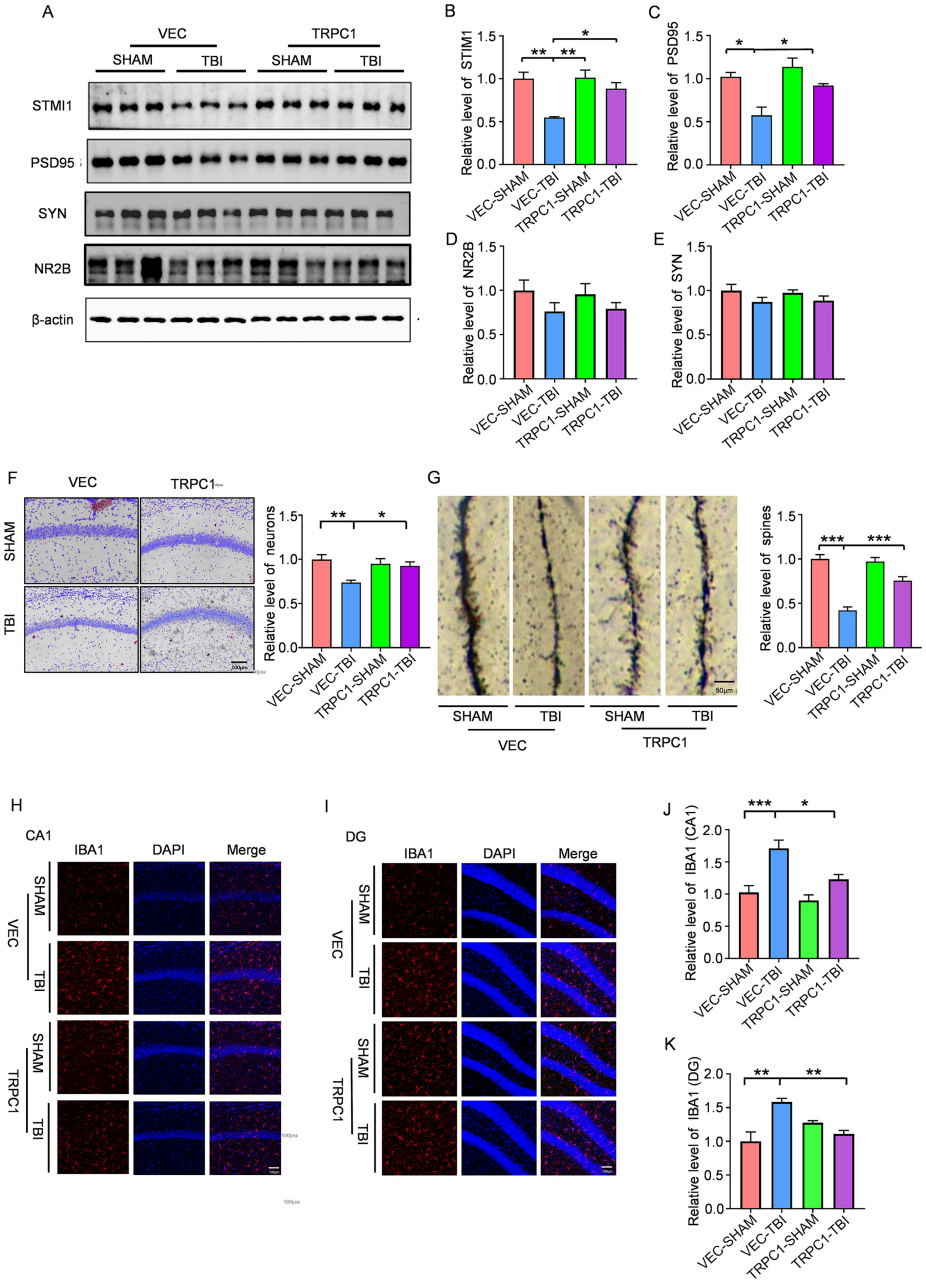
TRPC1 overexpression attenuates hippocampal inflammation and ameliorates neuronal loss and synaptic damage: **(A–E)** Western blotting showing the expression of STIM1 (*n* = 3 per group), PSD-95 (*n* = 3 per group), SYN (*n* = 3 per group), and NR2B (*n* = 3 per group) in hippocampus from the mice, post-viral transduction, with or without TBI. **(F)** Nissl staining of hippocampal regions showing neuronal integrity across experimental groups, *n* = 9 per group. **(G)** Golgi staining analysis of dendritic spine density in hippocampal neurons across experimental groups *n* = 9 per group. **(H)** Immunohistochemistry staining reveals the level of IBA1 in CA1. **(I)** Immunohistochemistry staining reveals the level of IBA1 in DG. **(J)** Quantification of IBA1 in CA1, *n* = 5 per group. **(K)** Quantification of IBA1 in DG, *n* = 4 per group. Data are analyzed by One-way ANOVA with Tukey’s multiple comparisons test, and represented as mean ± SEM; ***p < 0.001, **p < 0.01, *p < 0.05.

To further investigate whether TRPC1 overexpression alleviates depression-like behaviors by modulating neuroinflammation, the following experiments were conducted. Immunofluorescence staining revealed that, in the hippocampal CA1 and DG regions, the VEC-TBI group exhibited significantly increased IBA1 expression compared to the VEC-SHAM group, whereas the TRPC1-TBI group showed a decreasing trend in IBA1 expression relative to the VEC-TBI group, suggesting that TRPC1 overexpression mitigates post-TBI neuroinflammation (Figures 5H–K). Western blot analysis demonstrated that STIM1 expression was reduced in the VEC-TBI group compared to the VEC-SHAM group, along with decreased levels of synaptic proteins. In contrast, the TRPC1-TBI group exhibited elevated STIM1 expression and increased synaptic protein levels compared to the VEC-TBI group, indicating that TRPC1 overexpression improves synaptic function in the hippocampus of TBI-induced depressive mice (Figures 5A–E). Nissl staining revealed a decrease in neuronal number in the VEC-TBI group compared to the VEC-SHAM group, while the TRPC1-TBI group showed an increase in neuronal survival relative to the VEC-TBI group (Figure 5F), indicating that TRPC1 overexpression attenuates TBI-induced neuronal loss in the hippocampus. Golgi staining showed that dendritic spine density was reduced in the VEC-TBI group compared to the VEC-SHAM group, whereas the TRPC1-TBI group exhibited an increasing trend in spine density compared to the VEC-TBI group (Figure 5G), suggesting that TRPC1 overexpression enhances synaptic structural integrity in the hippocampus. These results collectively demonstrate that TRPC1 overexpression ameliorates depression-like behaviors in TBI mice by reducing hippocampal neuroinflammation, restoring synaptic function, preserving synaptic structural integrity, and preventing neuronal loss.

## 4 Discussion

This study provides the first evidence that TRPC1 may play a critical role in the pathogenesis of post-TBI depression and could serve as a potential therapeutic target. Our findings demonstrated that, at 4 weeks post-TBI, mice exhibited synaptic dysfunction, elevated neuroinflammation, and decreased TRPC1 expression in the hippocampal region, suggesting a possible involvement of TRPC1 in the development of post-TBI depression. Notably, TRPC1 overexpression effectively attenuated neuroinflammation in the hippocampus of TBI-depressed mice. Furthermore, it ameliorated neuronal loss, restored synaptic density, and improved synaptic function, ultimately leading to a significant alleviation of depressive-like behaviors.

Traumatic brain injury (TBI) frequently leads to physical disability and is often accompanied by severe neurodegenerative pathologies and neuropsychiatric sequelae (Howlett et al., 2022; Perry et al., 2016; Shrivastava et al., 2025; Zhu et al., 2024). Clinical studies indicate that up to 56% of TBI patients develop depressive symptoms within 10 weeks post-injury (Singh et al., 2018), and the risk of depression in TBI survivors remains nearly 11-fold higher than in healthy controls even 1 year after trauma (Choi et al., 2022). Pre-clinical investigations have corroborated these findings, demonstrating that rodents subjected to TBI exhibit increased susceptibility to depressive-like behaviors (Bahader et al., 2024; Iannucci et al., 2024; Shultz et al., 2020). Notably, studies examining the association between injury severity and depression reveal that moderate TBI induces more pronounced depressive behaviors in rodents (Carmichael et al., 2023a; Carmichael et al., 2023b; Popovitz et al., 2019). For instance, in a controlled cortical impact (CCI) model of moderate TBI, mice displayed significant depressive-like behavioral alterations at 30 days post-injury (Niziolek et al., 2020). Consistent with these reports, our study observed robust post-TBI depressive phenotypes in mice at 4 weeks after moderate CCI, accompanied by synaptic dysfunction in the hippocampus.

Mounting evidence indicates that synaptic dysfunction persists in the late phase following traumatic brain injury (TBI), mirroring the synaptic impairments observed in both clinical depression and animal models of the disorder. Postmortem studies of depressive patients have demonstrated significantly reduced protein expression of NMDA receptor subunits (GluN1, GluN2A, and GluN2B) and PSD95 in the prefrontal cortex and hippocampus compared to healthy controls (Feyissa et al., 2009; Sowa-Kućma et al., 2013). These findings are further supported by proteomic analyses in depression models, revealing decreased levels of GluN2A, GluN2B, PSD95, and synapsin1 in brain tissues (Lin et al., 2019; Shi et al., 2024; Zeng et al., 2023), suggesting that TBI-induced secondary synaptic damage may represent a key mechanism underlying post-TBI depression. Notably, a recent study showed that NLRP3 knockout attenuated pathogenic astrocyte activation, enhanced PSD95 expression, and significantly alleviated TBI-induced depressive-like behaviors (Miao et al., 2025). TRPC1, a calcium-permeable cation channel, is known to regulate diverse neuronal processes, including neurite outgrowth, synaptogenesis, neurotransmitter release, and synaptic plasticity (Kollewe et al., 2022; Skerjanz et al., 2024). While mGluR5-mediated TRPC1 overexpression has been reported to enhance synaptic function and improve neurological outcomes in mice (Lepannetier et al., 2018), the relationship between TRPC1 and post-TBI depression remains poorly understood. In our study, TRPC1 overexpression effectively restored synaptic function in the hippocampal region of TBI-depressed mice, preserved synaptic structural integrity, and reduced neuronal loss, highlighting its potential as a therapeutic target for mitigating synaptic deficits in post-TBI depression.

TBI induces an acute inflammatory response that often progresses into chronic neuroinflammation persisting for months to years (Karve et al., 2016; Simon et al., 2017a; Singhal et al., 2014), mirroring the well-documented elevation of pro-inflammatory cytokines (TNF-α, IL-1β, and IL-6) observed in both peripheral circulation and postmortem brain samples of depressed patients (Goldsmith et al., 2016). Preclinical evidence further supports this connection, as inhibition of IFN-β and CXCL10-mediated T-cell infiltration has been shown to attenuate neuroinflammation and depressive-like behaviors in a controlled cortical impact model (Sen et al., 2020). As a Ca^2+^-permeable ion channel activated by STIM1 during store-operated calcium entry, TRPC1 emerges as a critical regulator of inflammatory responses (Ambudkar, 2007; Qiu et al., 2021; Zhang et al., 2025). Previous studies have revealed that pharmacological inhibition of TRPC1/STIM1 with Brefeldin A or Tunicamycin increases NFkB phosphorylation and the production of IL-6, IL-1β, and TNF-α (Nascimento Da Conceicao et al., 2019). Conversely, febuxostat (FEB) has been reported to mitigate 5-FU-induced inflammatory damage through TRPC1/CHOP signaling (Abdelzaher et al., 2022). In this study, we overexpressed TRPC1 at the cellular level and treated cells with LPS to simulate heightened inflammation. Western blot and q-PCR analyses revealed that TRPC1 overexpression attenuated the pro-inflammatory effects of LPS, as indicated by reduced levels of inflammatory cytokines (IL-1β, TNF-α, and IL-6). Subsequent *in vivo* experiments involving lentivirus-mediated TRPC1 overexpression demonstrated diminished microglial activation in the hippocampal region 4 weeks after moderate traumatic brain injury (TBI). These findings suggest that TRPC1 overexpression may mitigate post-TBI depression-like behaviors by modulating neuroinflammatory responses.

In summary, our findings establish TRPC1 as a novel therapeutic target for post-TBI depression through its dual regulatory effects on neuroinflammation and synaptic plasticity.

## Data Availability

The raw data supporting the conclusions of this article will be made available by the authors, without undue reservation.
