# Supplementary material for: Role of TRPC1 in the pathogenesis of depression induced by traumatic brain injury
Source: Front Neurosci. 2026 Apr 16;20:1774265. doi: 10.3389/fnins.2026.1774265 (PMC13128399; doi:10.3389/fnins.2026.1774265)
Supplement: Supplementary file 1 [file Table_1.DOCX]

**Supporting Information**

**Role of TRPC1 in the Pathogenesis of Depression Induced by Traumatic Brain Injury**

Qi-Hang Pan, Lin-Han Li, Ming-Bo Fan, Yu Xia, Xiao-Long Liu, Zhen-Huan Chen, Fei Sun, Ting He, Zhou-Qiu Zhi, Meng-Zhu Li, Jun Li

**Table S1**: Neurological Severity Score (NSS) for mice.

| Task | Description | Points (success/failure) | |
| --- | --- | --- | --- |
| Exit circle | Ability and initiative to exit a circle of 30 cm diameter within 3 min | | 0/1 |
| Monoparesis/hemiparesis | Paresis of upper and/or lower limb of the contralateral side | | 0/1 |
| Straight walk | Alertness, initiative and motor ability to walk straight | | 0/1 |
| Startle reflex | Innate reflex; the mouse will bounce in response to a loud hand clap | | 0/1 |
| Seeking behavior | Physiological behavior as a sign of “interest” in the environment | | 0/1 |
| Beam balancing | Ability to balance on a beam of 7 mm width for at least 10 s | | 0/1 |
| Round stick balancing | Ability to balance on a round stick of 5 mm diameter for at least 10 s | | 0/1 |
| Beam walk: 3 cm | Ability to cross a 30-cm long beam of 3 cm width | | 0/1 |
| Beam walk: 2 cm | Same task, increased difficulty on a 2-cm wide beam | | 0/1 |
| Beam walk: 1 cm | Same task, increased difficulty on a 1-cm wide beam | | 0/1 |
| Maximal score |  | | 10 |

**Table S2.** Statistical analyses, related to Figures 1-5.

| Figure | Conditions | Analysis | P value | t or F value |
| --- | --- | --- | --- | --- |
| 1B | SHAM *vs*. TBI | Two-tailed unpaired *t* test | *p* < 0.0001 | t _(29)_ = 9.956 |
| 1C | SHAM *vs*. TBI (travel distance) | Two-tailed unpaired *t* test | *p* = 0.4186 | t _(29)_ = 0.8205 |
|  | SHAM *vs*. TBI (time in corner) | Two-tailed unpaired *t* test | *p* = 0.0002 | t _(29)_ = 4.218 |
| 1D | SHAM *vs*. TBI | Two-tailed unpaired *t* test | *p* < 0.0001 | t _(29)_ = 5.005 |
| 1E | SHAM *vs*. TBI | Two-tailed unpaired *t* test | *p* =0.0022 | t _(29)_ = 3.360 |
| 1F | SHAM *vs*. TBI | Two-tailed unpaired *t* test | *p* < 0.0001 | t _(29)_ = 17.39 |
| 1H | SHAM *vs*. TBI | Two-tailed unpaired *t* test | *p* = 0.08 | t _(4)_ = 2.333 |
| 1I | SHAM *vs*. TBI | Two-tailed unpaired *t* test | *p* = 0.0072 | t _(4)_ = 5.063 |
| 1J | SHAM *vs*. TBI | Two-tailed unpaired *t* test | *p* = 0.0113 | t _(4)_ = 4.439 |
| 1K | SHAM *vs*. TBI | Two-tailed unpaired *t* test | *p* < 0.0001 | t _(46)_ = 10.25 |
| 2B | SHAM *vs*. TBI | Two-tailed unpaired *t* test | *p* = 0.0165 | t _(4)_ = 3.969 |
| 2C | SHAM *vs*. TBI | Two-tailed unpaired *t* test | *p* = 0.0028 | t _(4)_ = 6.549 |
| 2D | SHAM *vs*. TBI | Two-tailed unpaired *t* test | *p* = 0.0807 | t _(4)_ = 2.325 |
| 2F | SHAM *vs*. TBI (CA1 region) | Two-tailed unpaired *t* test | *p* = 0.0017 | t _(6)_ = 5.367 |
|  | SHAM *vs*. TBI (CA3 region) | Two-tailed unpaired *t* test | *p* = 0.0014 | t _(6)_ = 5.558 |
|  | SHAM *vs*. TBI (DG region) | Two-tailed unpaired *t* test | *p* = 0.0002 | t _(6)_ = 7.772 |
| 2H | SHAM *vs*. TBI (CA1 region) | Two-tailed unpaired *t* test | *p* = 0.0354 | t _(6)_ = 2.703 |
|  | SHAM *vs*. TBI (CA3 region) | Two-tailed unpaired *t* test | *p* = 0.0035 | t _(6)_ = 4.658 |
|  | SHAM *vs*. TBI (DG region) | Two-tailed unpaired *t* test | *p* = 0.0378 | t _(6)_ = 2.655 |
| 3B | VEC-NV *vs*. VEC-LPS | One-way ANOVA with Tukey’s test | *p* = 0.0233 | F _(3, 20)_ = 6.524 |
|  | VEC-LPS *vs*. TRPC1-LPS | One-way ANOVA with Tukey’s test | *p* = 0.0020 | F _(3, 20)_ = 6.524 |
| 3C | VEC-NV *vs*. VEC-LPS | One-way ANOVA with Tukey’s test | *p* = 0.0022 | F _(3, 20)_ = 13.77 |
|  | VEC-LPS *vs*. TRPC1-LPS | One-way ANOVA with Tukey’s test | *p* < 0.0001 | F _(3, 20)_ = 13.77 |
| 3E | VEC-NV *vs*. VEC-LPS | One-way ANOVA with Tukey’s test | *p* = 0.0358 | F _(3, 20)_ = 5.171 |
|  | VEC-LPS *vs*. TRPC1-LPS | One-way ANOVA with Tukey’s test | *p* = 0.0068 | F _(3, 20)_ = 5.171 |
| 3F | VEC-NV *vs*. VEC-LPS | One-way ANOVA with Tukey’s test | *p* = 0.0071 | F _(3, 20)_ = 8.515 |
|  | VEC-LPS *vs*. TRPC1-LPS | One-way ANOVA with Tukey’s test | *p* = 0.0006 | F _(3, 20)_ = 8.515 |
| 3G | VEC-NV *vs*. VEC-LPS | One-way ANOVA with Tukey’s test | *p* = 0.0118 | F _(3, 20)_ = 6.318 |
|  | VEC-LPS *vs*. TRPC1-LPS | One-way ANOVA with Tukey’s test | *p* = 0.0115 | F _(3, 20)_ = 7.449 |
| 3H | VEC-NV *vs*. VEC-LPS | One-way ANOVA with Tukey’s test | *p* = 0.0091 | F _(3, 20)_ = 9.110 |
|  | VEC-LPS *vs*. TRPC1-LPS | One-way ANOVA with Tukey’s test | *p* = 0.0161 | F _(3, 20)_ = 9.110 |
| 3J | WT *vs*. TRPC1^ko^ | Two-tailed unpaired *t* test | *p* = 0.2396 | t _(10)_ = 1.250 |
| 3K | WT *vs*. TRPC1^ko^ | Two-tailed unpaired *t* test | *p* = 0.0486 | t _(10)_ = 2.244 |
| 3L | WT *vs*. TRPC1^ko^ | Two-tailed unpaired *t* test | *p* = 0.0151 | t _(10)_ = 2.926 |
| 3M | WT *vs*. TRPC1^ko^ | Two-tailed unpaired *t* test | *p* < 0.0001 | t _(10)_ = 7.925 |
| 3N | WT *vs*. TRPC1^ko^ | Two-tailed unpaired *t* test | *p* = 0.163 | t _(10)_ = 1.506 |
| 4C | VEC-SHAM *vs*. VEC-TBI | One-way ANOVA with Tukey’s test | *p* < 0.0001 | F _(3, 44)_ = 39.21 |
|  | TRPC1-SHAM *vs*. TRPC1-TBI | One-way ANOVA with Tukey’s test | *p* < 0.0001 | F _(3, 44)_ = 39.21 |
| 4D | VEC-SHAM *vs*. VEC-TBI | One-way ANOVA with Tukey’s test | *p* = 0.8719 | F _(3, 44)_ = 1.329 |
|  | TRPC1-SHAM *vs*. TRPC1-TBI | One-way ANOVA with Tukey’s test | *p* =0.3147 | F _(3, 44)_ = 1.329 |
| 4E | VEC-SHAM *vs*. VEC-TBI | One-way ANOVA with Tukey’s test | *p* < 0.0001 | F _(3, 44)_ = 15.03 |
|  | VEC-TBI *vs*. TRPC1-TBI | One-way ANOVA with Tukey’s test | *p* = 0.0086 | F _(3, 44)_ = 15.03 |
| 4F | VEC-SHAM *vs*. VEC-TBI | One-way ANOVA with Tukey’s test | *p* < 0.0001 | F _(3, 44)_ = 10.73 |
|  | VEC-TBI *vs*. TRPC1-TBI | One-way ANOVA with Tukey’s test | *p* = 0.0021 | F _(3, 44)_ = 10.73 |
| 4G | VEC-SHAM *vs*. VEC-TBI | One-way ANOVA with Tukey’s test | *p* = 0.0333 | F _(3, 44)_ = 7.112 |
|  | VEC-TBI *vs*. TRPC1-SHAM | One-way ANOVA with Tukey’s test | *p* = 0.2802 | F _(3, 20)_ = 1.329 |
| 5B | VEC-SHAM *vs*. VEC-TBI | One-way ANOVA with Tukey’s test | *p* = 0.0066 | F _(3, 8)_ = 10.22 |
|  | VEC-TBI *vs*. TRPC1-SHAM | One-way ANOVA with Tukey’s test | *p* = 0.0055 | F _(3, 8)_ = 10.22 |
|  | VEC-TBI *vs*. TRPC1-TBI | One-way ANOVA with Tukey’s test | *p* = 0.0321 | F _(3, 8)_ = 10.22 |
| 5C | VEC-SHAM *vs*. VEC-TBI | One-way ANOVA with Tukey’s test | *p* = 0.0127 | F _(3, 8)_ = 10.37 |
|  | VEC-TBI *vs*. TRPC1-SHAM | One-way ANOVA with Tukey’s test | *p* = 0.0033 | F _(3, 8)_ = 10.37 |
|  | VEC-TBI *vs*. TRPC1-TBI | One-way ANOVA with Tukey’s test | *p* = 0.0463 | F _(3, 8)_ = 10.37 |
| 5D | VEC-SHAM *vs*. VEC-TBI | One-way ANOVA with Tukey’s test | *p* = 0.4128 | F _(3, 8)_ = 1.319 |
|  | VEC-TBI *vs*. TRPC1-TBI | One-way ANOVA with Tukey’s test | *p* = 0.9967 | F _(3, 8)_ = 1.319 |
| 5E | VEC-SHAM *vs*. VEC-TBI | One-way ANOVA with Tukey’s test | *p* =0.3788 | F _(3, 8)_ = 1.421 |
|  | VEC-TBI *vs*. TRPC1-TBI | One-way ANOVA with Tukey’s test | *p* = 0.9971 | F _(3, 8)_ = 1.421 |
| 5F | VEC-SHAM *vs*. VEC-TBI | One-way ANOVA with Tukey’s test | *p* = 0.0026 | F _(3, 32)_ = 5.829 |
|  | VEC-TBI *vs*. TRPC1-SHAM | One-way ANOVA with Tukey’s test | *p* = 0.0163 | F _(3, 32)_ = 5.829 |
|  | VEC-TBI *vs*. TRPC1-TBI | One-way ANOVA with Tukey’s test | *p* = 0.0391 | F _(3, 76)_ = 5.829 |
| 5G | VEC-SHAM *vs*. VEC-TBI | One-way ANOVA with Tukey’s test | *p* < 0.0001 | F _(3, 76)_ = 36.22 |
|  | VEC-TBI *vs*. TRPC1-TBI | One-way ANOVA with Tukey’s test | *p* < 0.0001 | F _(3, 76)_ = 36.22 |
| 5J | VEC-SHAM *vs*. VEC-TBI (CA1 region) | One-way ANOVA with Tukey’s test | *p* = 0.0001 | F _(3, 16)_ = 12.56 |
|  | VEC-TBI *vs*. TRPC1-TBI (CA1 region) | One-way ANOVA with Tukey’s test | *p* = 0.0172 | F _(3, 16)_ = 12.56 |
| 5K | VEC-SHAM *vs*. VEC-TBI (DG region) | One-way ANOVA with Tukey’s test | *p* = 0.0012 | F _(3, 12)_ = 10.05 |
|  | VEC-TBI *vs*. TRPC1-TBI (DG region) | One-way ANOVA with Tukey’s test | *p* = 0.0060 | F _(3, 12)_ = 10.05 |
